# The movement ecology of the Mauritian flying fox (*Pteropus niger*): a long-term study using solar-powered GSM/GPS tags

**DOI:** 10.1186/s40462-019-0156-6

**Published:** 2019-04-15

**Authors:** Ryszard Z. Oleksy, Charles L. Ayady, Vikash Tatayah, Carl Jones, Paul W. Howey, Jérémy S. P. Froidevaux, Paul A. Racey, Gareth Jones

**Affiliations:** 1 School of Biological Sciences, Life Sciences Building, University of Bristol, 24 Tyndall Avenue, Bristol, BS8 1TQ UK; 2 Department of Zoology, The George S. Wise Faculty of Life Science Tel Aviv University, P.O. Box 39040, Tel Aviv, Israel; 3 Ecosystem Restoration Alliance Indian Ocean, No. 7 Site and Services, Circonstance, St. Pierre, Mauritius; 4 Durrell Wildlife Conservation Trust, Les Augrès Manor, Trinity, Channel Islands JE3 5BP Jersey; 5 Mauritian Wildlife Foundation, Grannum Road, Vacoas, Mauritius; 6 Microwave Telemetry, Inc, 8835 Columbia, 100 Parkway, Suites K & L, Columbia, MD 21045 USA; 7 Centre for Ecology and Conservation, School of Biosciences, University of Exeter, Penryn Campus, Penryn, TR10 9FE UK

## Abstract

**Background:**

Flying foxes (Chiroptera: Pteropodidae) are large bats that often roost in the sun, hence solar-powered GPS/GSM devices can track their movements over extended periods. The endemic Mauritian flying fox (*Pteropus niger*) has recently been subjected to large-scale culling because of perceived damage to commercial fruit, and a consequent reduction in numbers of > 50% since 2015 resulted in its IUCN Red List Status being up-listed to Endangered. Determining its movements will be important for management and conservation, for understanding potential responses to environmental change, and for understanding population admixture.

**Methods:**

Twelve bats were tagged with solar-powered GPS/GSM devices in 2014–2016. Tags remained active for up to almost a year (maximum 359 days: average 139 days (males) and 93 days (females)), providing some of the longest-term data on the movement ecology of bats yet obtained. Eight bats were probably hunted illegally, highlighting the scale of unauthorised persecution.

**Results:**

Males travelled on average 9 km each night, females 6 km. The nightly distance covered by adults of both sexes was higher in winter than in summer, though the opposite pattern occurred for immature males. These differences are probably related to seasonal changes in fruit availability (adults) and to dispersal by immature males. The maximum distance covered during one night was > 92 km. Home ranges of males averaged 74,633 ha, females 31,072 ha. Core foraging areas averaged 2222 ha for males, 1364 ha for females. Fifty roosts were identified, mainly in forest fragments. As the bats disperse seeds of native plants that form forest canopies, conservation of the bats will potentially maintain and enhance native forest cover, in turn providing roosting sites for the bats.

**Conclusions:**

Solar-powered GSM tagging provides unprecedented potential for understanding the movement ecology of flying foxes. Mauritian flying foxes often move between the few remnant native forest fragments, which remain important for their conservation, and have potentially important roles in seed dispersal. Their nomadic movement fits with their panmictic genetic structure. Although their ability for long distance movements, sometimes over short timescales, permits rapid responses to local threats and environmental change, being restricted to Mauritius renders the bats extremely vulnerable to intense culling.

**Electronic supplementary material:**

The online version of this article (10.1186/s40462-019-0156-6) contains supplementary material, which is available to authorized users.

## Background

Movement of individual organisms is one of the most fundamental features of life on earth and a major component of ecological and evolutionary processes [1]. Animals move for many reasons such as the search for resources, predator or competition avoidance and to be near conspecifics for mating and other social interactions [2]. Movement plays a pivotal role in shaping biodiversity patterns across spatiotemporal scales. It affects biodiversity directly and indirectly by determining how species are distributed and how they interact [3, 4]. Movement patterns can also help understand how animals may respond to threats and environmental change [5], and provide a framework for understanding population genetic structure [6], with unrestricted movement potentially giving rise to a lack of genetic structure, leading to panmixia over large spatial scales.

The most common types of movements involve foraging, dispersal and migration [7, 8], generally with major differences in spatiotemporal scales [8]. Foraging is usually undertaken within a home range, while dispersal refers to movements away from the place of birth towards another location for reproduction [8]. Migratory movements often follow seasonal fluctuations in resource availability, and can cover thousands of kilometres [8, 9]. However, spatial and temporal variability in environmental conditions may affect all types of movement across all scales, from local to global, creating new opportunities for the evolution of movement parameters [2, 10]. Whether or not a species’ movement parameters are adaptive in the changing landscape will depend on the rate of landscape change relative to the rate at which the species can evolve in response to that change [2]. The ability to move rapidly away from unsuitable conditions, or from threats can potentially have considerable adaptive value.

Understanding the movement ecology of frugivores is important given their ability to disperse seeds, often over long distances. Forested habitats are often fragmented as a consequence of deforestation, and seed dispersers play important roles in dispersing seeds among forest fragments [11]. Seed dispersal and pollination are the most threatened processes involving plant regeneration, and are recommended as priorities in the conservation of forests on a global scale [12]. Flying foxes are especially important long-distance seed dispersers in tropical and subtropical forested landscapes [13–15], and can also enhance the germination success of seeds that pass through their digestive tracts – they are potentially important for promoting forest regeneration [14].

The Mauritian flying fox (*Pteropus niger*) is a medium-sized frugivorous bat endemic to the Mascarene Islands. It was once distributed throughout the archipelago, although it is now restricted to the island of Mauritius (with records of several individuals present on nearby Réunion island) as a result of habitat destruction and hunting [16–19]. The species has a disproportionately large role as a seed disperser in Mauritius. It disperses the seeds of most of the woody plants in the native remnant forests on the island. *Pteropus niger* disperses the seeds of many endemic plant taxa, and the seeds of tall tree species that fill functionally important roles in forest canopies [20]. *Pteropus niger* is therefore playing a key role in ecosystem services in Mauritius, which forms part of a biodiversity hotspot [21].

Studies of the ecology and conservation status of the species were prioritised in an IUCN action plan [22], and important studies have been conducted on diet [20, 23]. The island habitat limits its range and the species is significantly affected by tropical cyclones, which can decimate island populations of flying foxes [9, 24, 25]. In common with many bat species, *P. niger* produces only a single offspring per year (rarely two) so recovery after a population crash is slow [26].

In 2013, the IUCN down-listed the species from Endangered to Vulnerable due mainly to earlier increases in numbers [26]. In response to pressure from fruit growers, the Government of Mauritius authorised a national cull of over 30,000 individuals in 2015 and an additional 10,000 in late 2016 due to the damage they are perceived to cause to mango (*Mangifera indica*) and litchi (*Litchi chinensis*) fruit [26, 27]. A recent study confirmed that fruit loss due to consumption by *P. niger* can be considerable (about a quarter of all fruits monitored overall, with birds damaging a further 6% of monitored fruits [28]), although such damage can be reduced greatly by covering trees with netting [28]. In 2016 the population of *P. niger* on Mauritius was estimated at only 62,500 ± 7%, (a decline of 50% since 2015) and given the mass culling, on-going illegal hunting, habitat degradation by invasive species and vulnerability to cyclones, the IUCN up-listed the species to Endangered once again [26]. Despite this up-listing, a further cull was initiated in 2018.

In this paper we present the first study of the movement of a fruit bat using transmitters equipped with a Global Positioning System (GPS) using the Global System for Mobile Communications (GSM). The tags were solar powered, and because the bats roost on trees in the open, could potentially transmit data over long time periods, though with a limited number of fixes per night. Because spatial data were transmitted to mobile phone networks, it was not necessary to recapture bats to download GPS fixes. Previously several studies have used GSM technology successfully on birds [29].

Little is known about the movement pattern of these bats apart from inferences that can be made from a genetic study, which concluded that the Mauritian population of *P. niger* is likely to be panmictic, with moderate to high levels of gene flow occurring among colonies distributed across the island [19]. Despite the high rate of deforestation of Mauritius and the highly altered and fragmented landscape with extensive plantations of sugar cane and commercial fruit, and increased levels of urban development [17], the bats survive in relatively large numbers. We tested the hypothesis that bats are ‘nomadic’ and able to move across the island within one night. We hypothesised that the bats’ panmictic genetic structure may be a consequence of long-distance movements over the island, resulting in a lack of population differentiation. We tested whether movement patterns varied according to sex, age and season. The mating season begins around May, with females giving birth at colonies between August and December when commercial crops such as lychees and mango are fruiting in orchards and gardens [26]. We also aimed to relate movement patterns to the species’ ability to respond to rapid environmental change and threats.

## Methods

The study took place between December 2014 and October 2016. The bats were captured at different locations around the island (Tab. 1) using mist nets (60 × 60 mm mesh; Ecotone, Poland) placed in tree canopies. No bats were captured close to known roosts or close to fruiting orchards. Seven out of 12 bats were tagged outside of the commercial fruiting season. Only two bats were captured in areas where fruiting orchards were nearby, at the end of the litchi fruiting season. Following capture, bats were weighed (Salter Spring Balance Scale (FKA Brands, Tonbridge, UK): capacity 2 kg, accuracy 10 g) and their forearm lengths were measured to the nearest 0.5 cm using a measuring tape. The bats weighed 550–800 g (Table 1), so the tags were always < 5.5% of the bats’ body mass. The reproductive status of males was assessed from external examination of scrotal testes [30]. Female reproductive status was determined by examination of nipples. Small nipples with no evidence of previous suckling meant that females were nulliparous and immature [31]. Suckling resulted in the nipples becoming dark keratinised protuberances, indicative of adulthood and sexual maturity [30].

**Table 1:** Twelve bats tagged with GPS/GSM devices; F-Female, M-Male; SM-Sexually Mature (M-large testes, F- previously had pups), SI- Sexually Immature (M-no visible testes; F- had not produced any pups yet)

Sex	F	F	F	F	F	F	M	M	M	M	M	M
Body mass (g)	620	610	550	560	600	760	600	680	600	550	800	550
Forearm (mm)	155	153	135	145	145	150	155	150	160	150	160	150
Sexual maturity	SM	SM	SI	SI	SM	SM	SI	SM	SI	SI	SM	SI
Capture location	Blue Bay	Blue Bay	Ferney	Verdun	Queen Victoria	Black River	Calebasses	Beaux Songes	Black River	Arsenal	Black River	Black River
Lat/long	20.4421°S 57.7190°E		20.3679°S 57.6978°E	20.2343°S 57.5547°E	20.2195°S 57.7133°E	20.3708°S 57.3949°E	20.1174°S 57.5561°E	20.2774°S 57.4176°E		20.1034°S 57.5391°E		
Tag ID	464	469	470	474	4651	4653	466	468	471	472	4652	473
Period	Feb-May 2015	Dec 2014-Mar 2015	Apr- Oct 2015	Sep 2015-Mar 2016	Apr-Sep 2015	June-Oct 2015	Jan-May 2015	Dec 2014-Apr 2015	Sep 2015-Sep 2016	Jan-Feb 2015	Dec 2015-Jan 2016	Sep 2015-Feb 2016
Days	47	98	75	123	114	98	124	128	359	51	26	147
Fixes	234	1108	342	799	993	387	946	1614	4934	585	134	1174

Latitude and longitude are given once for each capture site

Bats were tagged with GPS/GSM devices (ca. 30 g) equipped with miniature solar panels (Microwave Telemetry, Columbia, MA, USA). The tags were attached using leather collars around the neck (with finger-wide space to ensure it was not too tight) and secured with a small bolt and a nut. The leather exposed to changing temperatures and humidity would eventually degrade and fall off, as happened with five tags. The transmitter was positioned between the shoulders of the bat. Prior to the study, the tag attachment method was tested on a captive population of Mauritius fruit bats for a period of two weeks. The tags did not affect behaviour in any obvious way and thus were considered safe to use. The tags stayed on bats for less than a month to almost a year (Table 1). Two tagged bats were observed roosting and no sign of discomfort was noticed. The data were transmitted every morning directly to Microwave Telemetry by email using GSM services (Emtel Ltd., Ebene, Mauritius) and then circulated by email to the research team. The fixes were collected at different rates depending on the battery charge, which in turn depended on the amount of solar radiation (see Results for fix return rates).

The tracks of bats were viewed using Google Earth (Google, Mountain View, CA, USA) and ArcMAP 10 (Esri, Redlands CA, USA) software. We constructed minimum convex polygons (MCPs) to determine home ranges of individual bats. MCPs contain commuting routes used by bats to reach foraging areas and to return to the roost. To better define foraging areas, we analysed utilisation distribution discontinuities, plotting the MCP area against percentage inclusion of fixes [32]. Outlying fixes can then be excluded [33, 34] and cluster cores can be used to better indicate where animals concentrate their foraging time. The method is appropriate for animals such as bats that focus foraging activities in relatively small areas, and which move rapidly between foraging sites [35, 36]. Home range analyses were performed using Ranges 7 (Anatrack Ltd., Dorset, UK [37]).

All statistical analyses were conducted using R v3.4.1 [38]. We tested whether nightly movement of *P. niger* differs between sex (male vs. female), age (sexually mature vs. immature), and season (summer (October–March) vs. winter (April–September)) by fitting a series of generalised linear mixed models (GLMMs, function *glmer* in “lme4” package [39] with negative binomial distributions to handle overdispersion. Data were beforehand rounded to the nearest integer for use in GLMMs. The interactions between sex, age, and season were incorporated as fixed effects into the models while bat ID was included as random effect to account for pseudo-replication. Model fit was verified and validated using the “DHARMa” package [40]. We then conducted model selection using the *dredge* function (“MuMIn” package [41]) to identify the most parsimonious model. All possible combinations of variables within the full model were ranked using the second order Akaike information criterion (*AICc* [42]). The model that included the three-way interaction between sex, age, and season was identified as the most parsimonious one (*ΔAICc* of second best model > 4). We finally used the “lsmeans” package [43] to undertake post hoc contrast tests while correcting for multiple comparisons using the Tukey method.

## Results

### Tagged bats

In total 12 bats (six males and six females) were tagged with GPS/GSM transmitters (Table 1). Females that were obviously pregnant, or could have dependant young, were not tagged. The average body mass of females was 617 (± 76 SD) g and of males 630 (± 96) g. A total of 9387 fixes was collected over 835 days for males and 3863 fixes over 555 days for females. The tags remained on male bats for an average of 139 (± 118 SD) days and for 93 (± 28 SD) days on females. The male monitored for the longest time provided data for 359 days, and for females maximum monitoring time was  123 days. On average the tags provided 10 (± 3 SD) fixes per 24 h for males and 7 (± 3 SD) fixes per 24 h for females.

Data transmission stopped once the tag dropped off and had no access to sunlight, or when the bat was presumed killed by local people (which happened on five occasions). On two of the five occasions the tag was retrieved and re-used after a bat was killed and the tag discarded in an open field. Fortunately, these two tags had access to sunlight and kept transmitting their position, which enabled us to find them. On three occasions data transmission ended suddenly during early evening while bats was feeding on a commercial fruiting tree. The bats were probably killed and the tag destroyed or thrown away with no access to sunlight. Hence eight of 12 bats disappeared in conditions that suggested they were hunted. The rest of the tags stopped transmitting in remote forested areas. The sites were visited based on the last GPS coordinate received but we were unable to locate the tags because of inaccessible terrain. On these occasions the tag may have remained attached, but failed to transmit data.

### Tracking results

Females travelled on average 6.06 (± 4.04 SD) km per night while males travelled 9.38 (± 5.16 SD) km per night. The longest distance travelled within one night by a male bat was 92.92 km in March and for a female bat 79.19 km in April. On a monthly basis, females travelled an average of 140.43 (± 90.1 SD) km and males 253.84 (± 164 SD) km. Average nightly distances travelled by each bat varied from over 20 km to < 5 km a night.

**Fig. 1:**
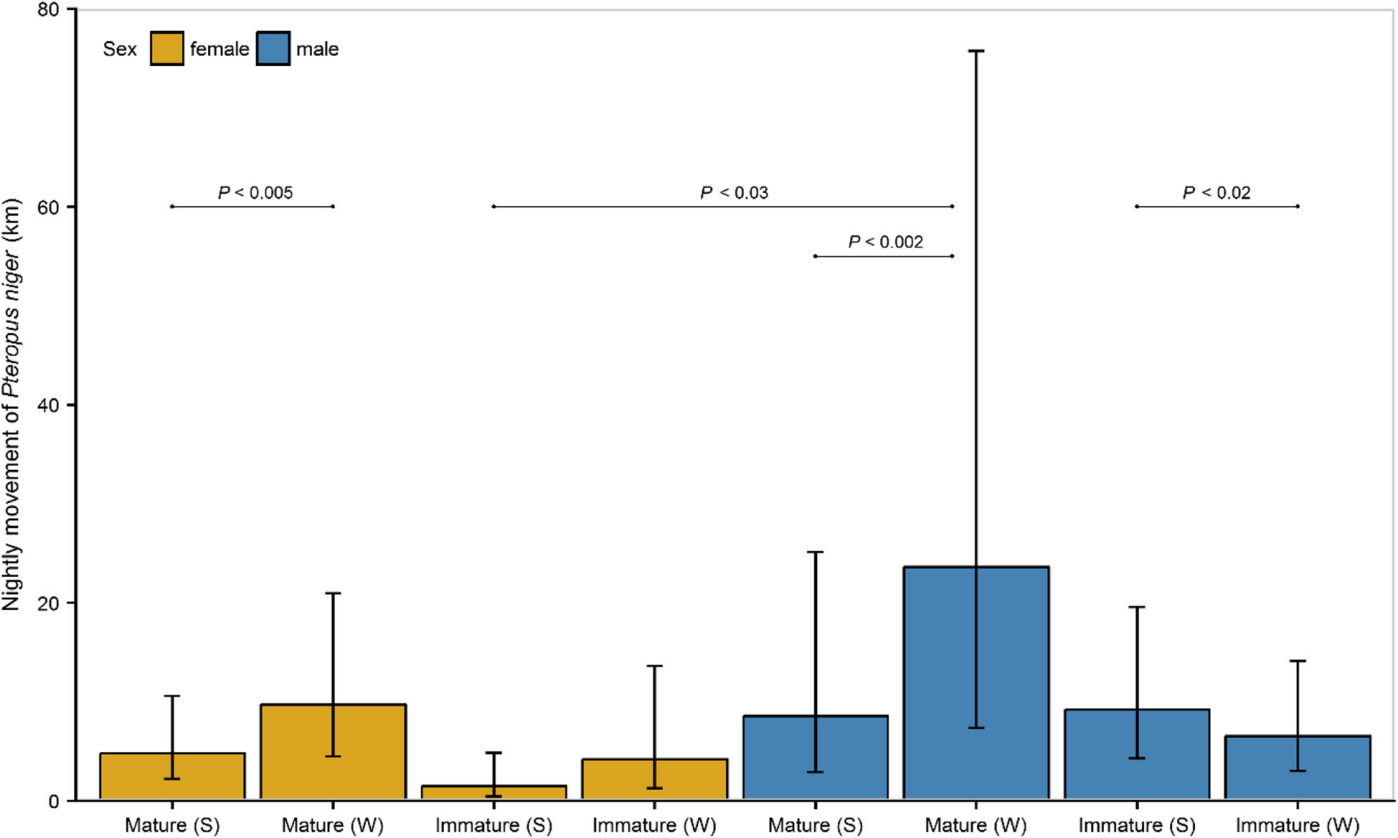
Predicted means and associated 95% confidence intervals of nightly movement of females and males *Pteropus niger* during summer (S) and winter (W), respectively. Model predictions arise from the most parsimonious GLMM that included three-way interactions between sex, age, and season. *P*-values adjusted for multiple comparisons are given for the statistically significant pairwise comparisons (*P*_adj_ < 0.05)

When investigating the effects of sex, age, and season on nightly movement of *P. niger*, the most parsimonious model (Additional file 1: Table S1) included interactions among all parameters. Post hoc contrast tests (Additional file 1: Table S2) revealed that adults of both sexes move significantly greater distances during winter than in summer while nightly movement of immature males were greater in summer than in winter (Fig. 1).

**Fig. 2:**
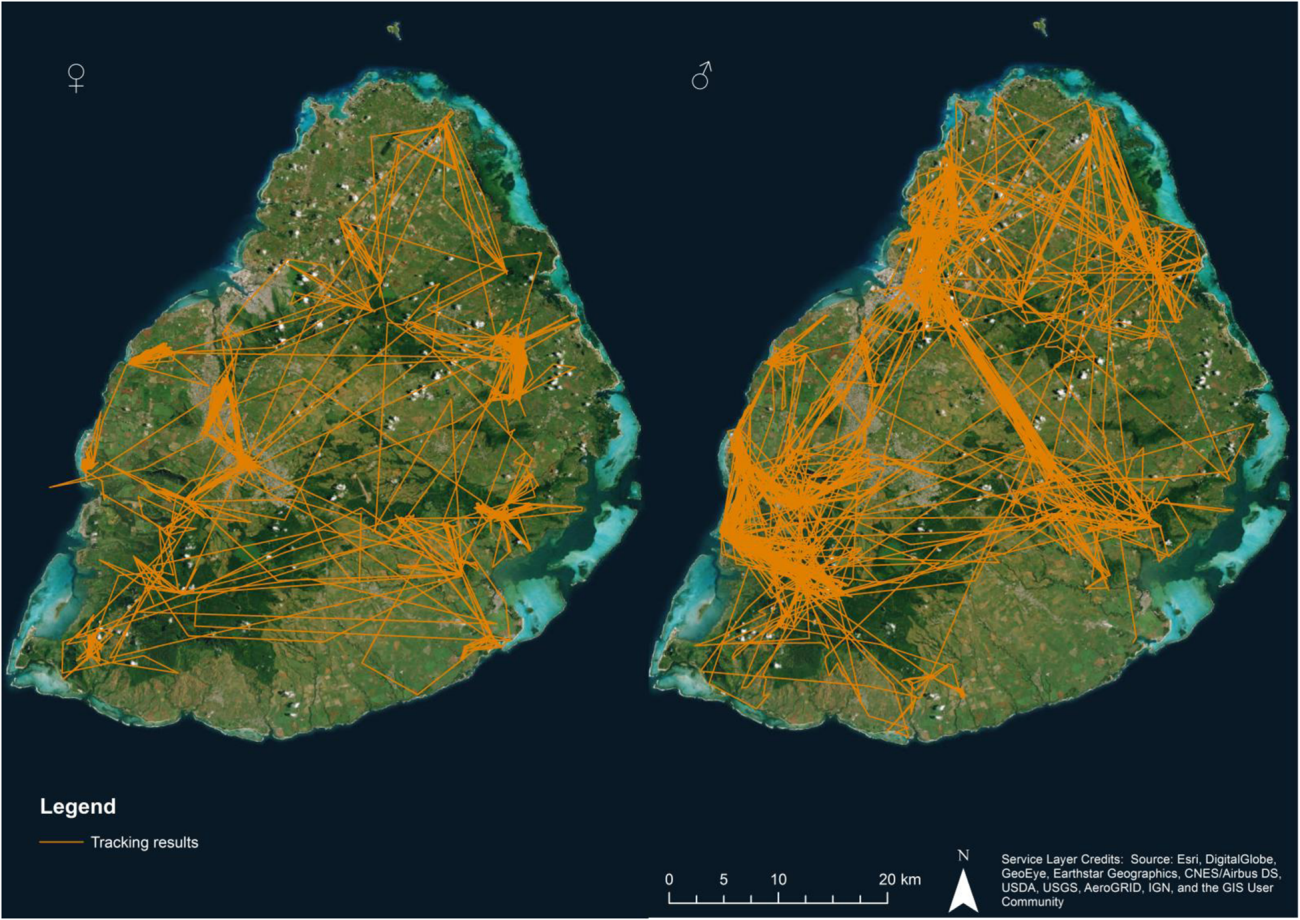
Visualised tracking results (orange lines) for all tagged females (left) (*N* = 6) and males (right) (*N* = 6) over the whole period of the study (December 2014–September 2016). The main forested areas are shown in dark green

**Fig. 3:**
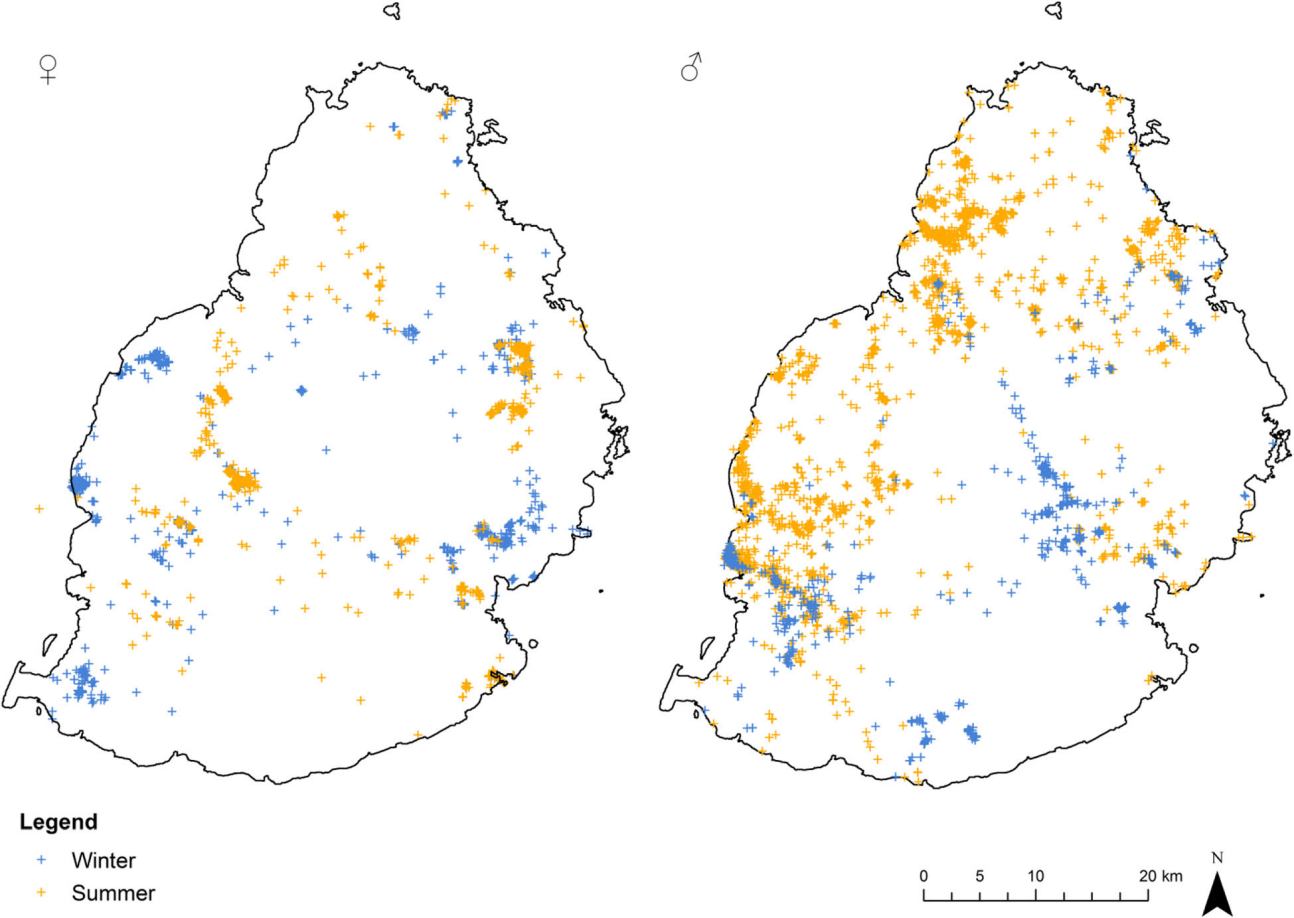
Fixes recorded for females (*N* = 6: left) and males (*N* = 6: right) during winter (April–September, (blue)) and summer (October–March, (yellow))

The movement pattern of females and especially males (Fig. 2) is concentrated in four main forested areas: the Moka mountain range in the northwest, Bras d’Eau National Park in the northeast, Black River Gorges National Park in the southwest and the Bambou mountain range in the southeast. These areas host the major bat roosts in Mauritius. Our visits in the north and southwest areas during May–April and October–November to the roosting sites showed large fluctuations in bat numbers. For example the roost in Pamplemousses Botanical Garden in the north varied from over 3000 individuals in early November to around a 100 in April/May. A major roost in the Black River Gorges National Park in the southwest contained over 3000 bats in April while in November only around 300 bats were seen. We were not able to monitor roosts in the southeast due to access difficulties. However differences in movement patterns occurred between summer (October–March) and winter (April–September). In general the seasonal changes in areas occupied were more evident in males than in females (Fig. 3). The males spent more time in the south of the island during the winter and dispersed over northwest parts in the summer.

### Home ranges

**Fig. 4:**
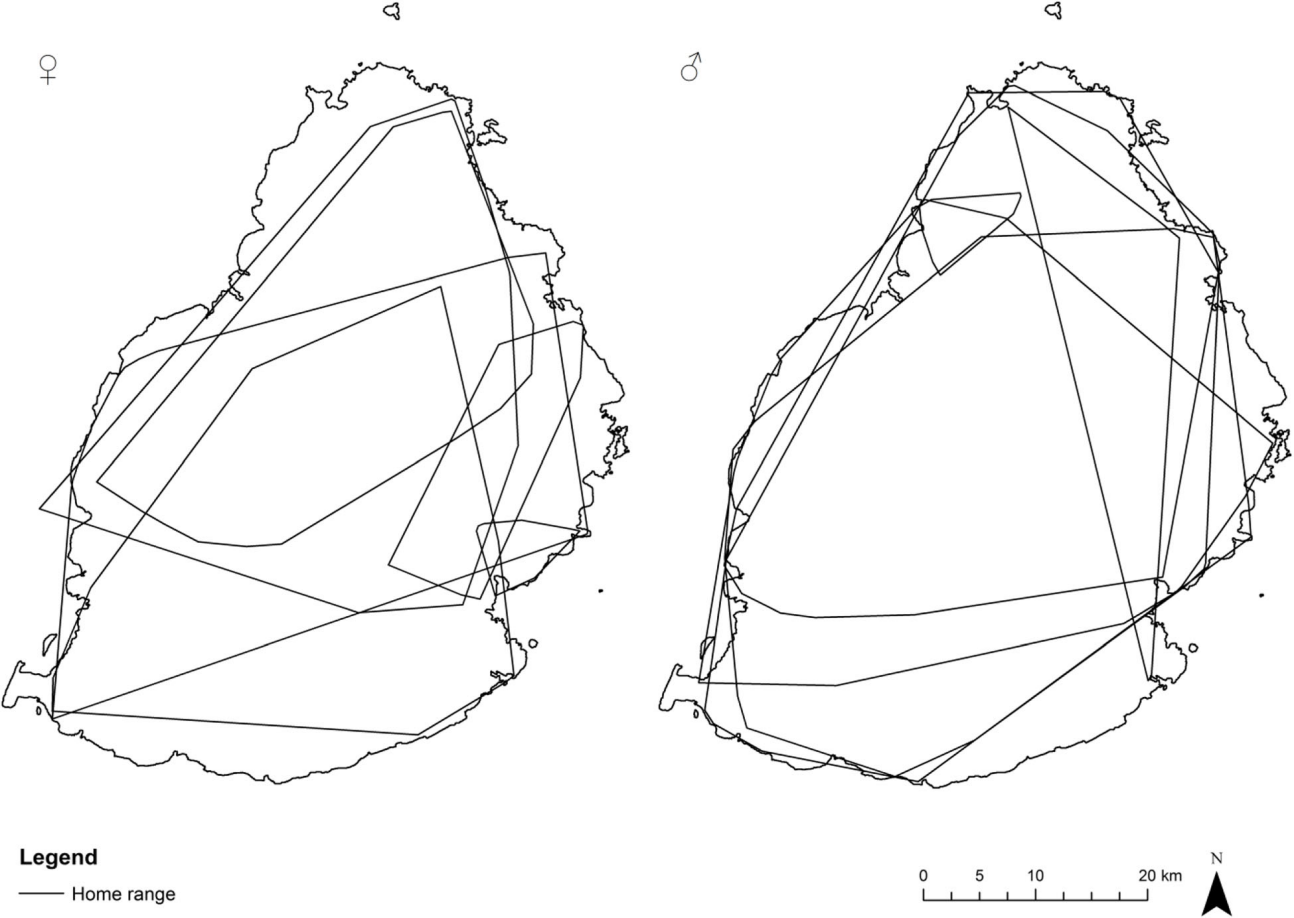
Home ranges of females (*N* = 6: left) and males (*N* = 6: right) presented as minimum convex polygons

The home ranges for each bat (Fig. 4) varied between 1243 ha to 149,924 ha for males with a mean of 74,633 ha (± 57,302 SD); and from 1500 ha to 72,511 ha for females (mean 31,072 ha± 27,374 SD). An independent samples T-test showed that there is no significant difference between male and female home range size (t = 1.68; df = 10; *p* = 0.054), although the difference was very close to significance. The trend could be due in part to males being tagged for longer periods on average than females.

**Fig. 5:**
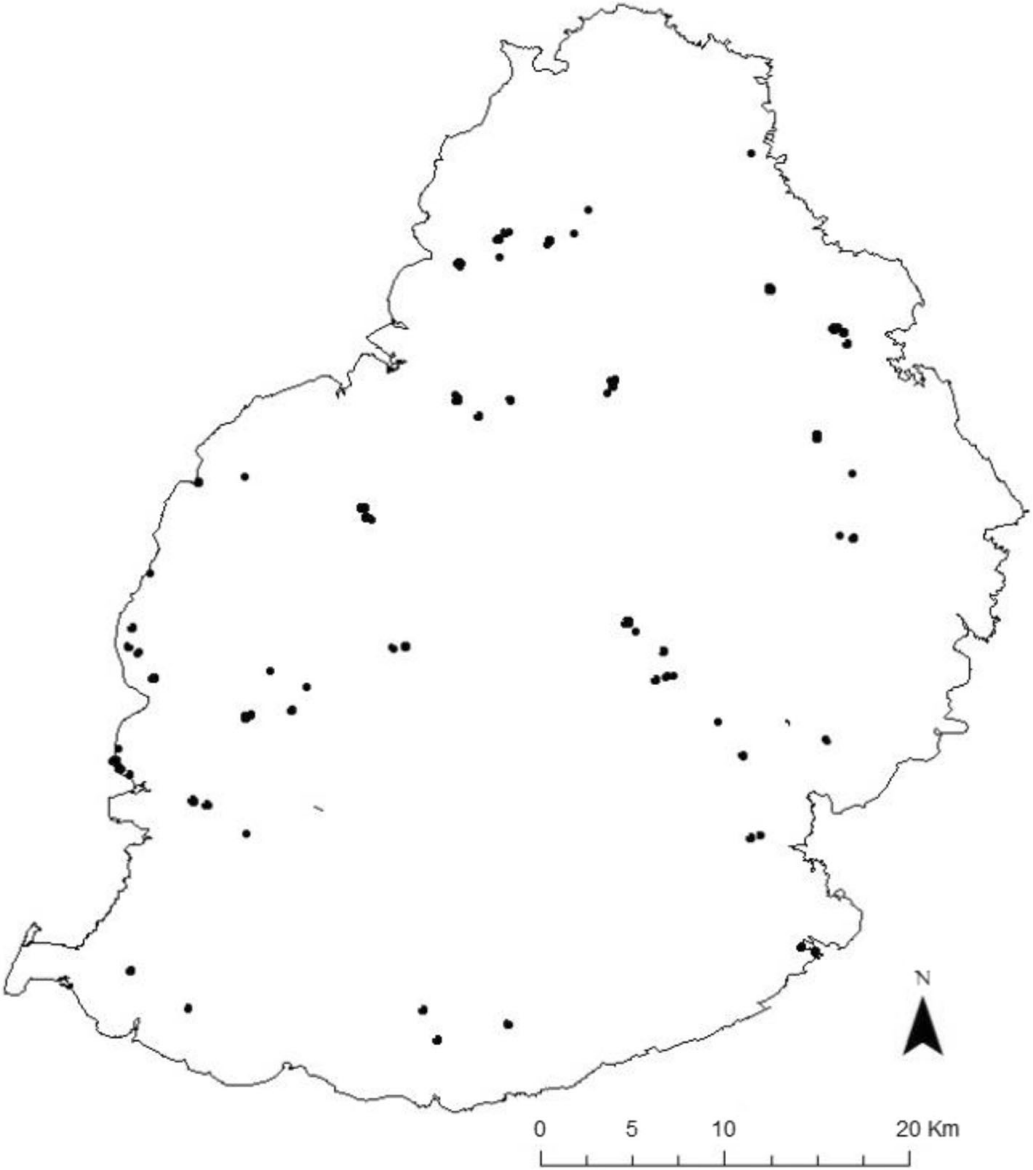
Map of *P. niger* roosting sites used by all tracked bats (*N* = 12)

From all the roosting data (fixes collected between 07.00 h and 16.00 h) 50% cluster core polygons were extracted to remove single fix locations and outlying fixes. That resulted in over 50 roosting sites used by *P. niger* during the whole study (Fig. 5). Roost overlap among individuals was small with an average of 12.3% (± 10.47 SD) overlap among all of the tracked individuals over the duration of the study, meaning that tagged bats rarely roosted together.

**Fig. 6:**
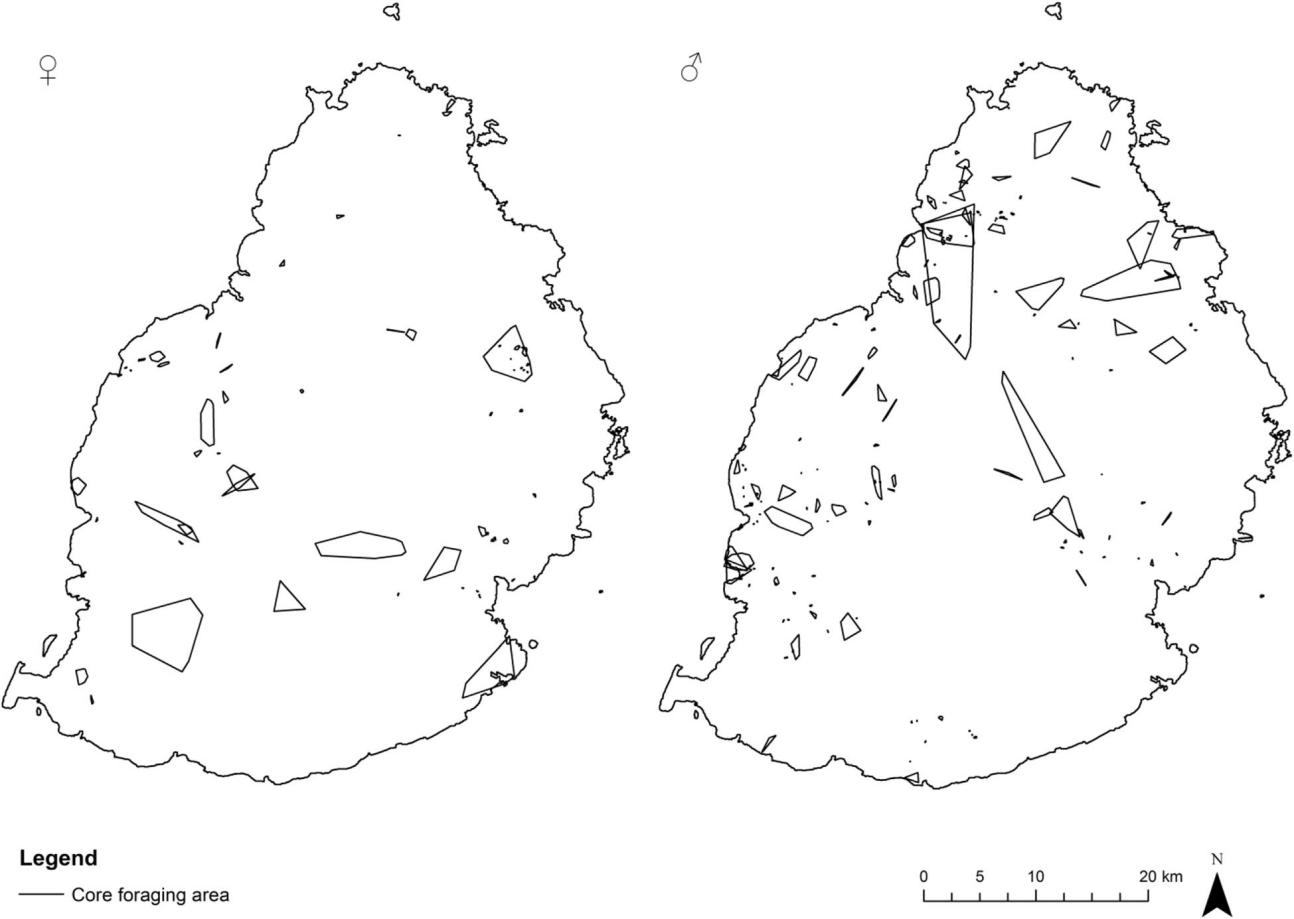
Core foraging area of females (*N* = 6: left) and males (*N* = 6: right) presented as 90% cluster core polygons

To define core foraging areas of the bats (Fig. 6), analysis of utilisation distribution discontinuities were used [23]. The analysis indicated that on average the bats used 90% cluster cores as presumed foraging areas. The remaining 10% would have caused a disproportionate increase in areas utilised by the bats, since it included the paths they used to travel to and from the feeding sites.

Core foraging areas covered a mixture of habitats and varied among individual bats from 33 ha to 9421 ha for males with an average of 2222 ha (± 3613 SD) and from 5392 ha to 1623 ha for females with an average of 1364 ha (± 2061 SD). An independent samples T-test showed no significant difference between the sizes of male and female core foraging areas (t = 0.505; df = 10; *p* = 0.413). The overlap between the foraging areas of individuals was generally small with an average of 15.2% (± 21.0 SD) overlap.

## Discussion

GSM tagging offers exciting new opportunities for studying the movement ecology of bats, especially for large flying fox species that roost in open areas such as in trees, where sunlight can recharge solar powered batteries. This recharging potential, coupled with access to GPS fixes via a mobile phone network, allows an understanding of the movement ecology of large bats over long time periods, providing insights into both short- and long-term use of space in ways that has hitherto not been possible. The return rate of fixes allowed nightly movements to be determined reliably, but was not sufficient to identify all foraging sites used in a night. Our study shows that *Pteropus niger*, despite living on a relatively small island, can cover large distances (the length of the island) sometimes in a single night, and that bats can move over much of the island in relatively short time periods. The bats can potentially respond rapidly to sudden changes in their environment by moving over large distances in short time periods. This nomadic movement pattern is consistent with the finding that *P. niger* show no obvious population structure on Mauritius [19].

*Pteropus niger,* along with other *Pteropus* species, is tree-roosting and thus dependent on vegetation cover [44]. Although 25% of Mauritius is covered by forest fragments, only about 5% of the land cover consists of native forest, of which only a third has > 50% native canopy cover [26, 45]. Given that *P. niger* uses forest extensively for roosting, and that it is a key disperser of the seeds of native tree species that form the forest canopy [20], conservation of the bats will promote the dispersal of canopy trees, whose growth will in turn provide suitable roosting sites for the bats. By choosing a suitable roosting site bats gain mating opportunities, reduced commuting costs to foraging sites, increased social interaction, and protection from adverse weather and predators [46–48]. In our study the bats used over 50 roosting sites distributed across the island, while recent NPCS reports highlight over 80 roosting sites (NPCS, pers. comm.).

Flying foxes are also highly mobile and their wing morphology allows them to track resources over large distances (5–80 km) and among scattered forest fragments [49–53]. In the present study the longest distance covered during one night by *P. niger* was over 92 km for a male and nearly 80 km for a female. Because Mauritius is a small island of only 1865 km^2^ with native forest cover of less than 100 km^2^, or 5% of the land area [17, 45, 54], bats are able to move across the island and between the forest fragments within one night. Therefore, Mauritius has no physical boundaries that impede the bats for tracking food resources or finding a mate, and the whole island is the potential home range of individual bats*.*

Movement between forested areas may be associated with finding a suitable roost or decreasing the distances travelled in search of food. Although *P. niger* roosts in public places such as the botanic garden in Pamplemousses and at Jardin de la Compagnie (a park in the capital city of Port Louis), the remnant forest patches provide much safer environments for roosting. Roosting in public places is a recent phenomenon (perhaps becoming prevalent only in the last decade) and could be the consequence of relatively high population levels (prior to culling), reduced levels of hunting prior to the cull, or to the loss of resources in remnant forest patches. Ultimately, the remaining forest fragments are crucial for the conservation of the species. Additionally, the scattered roosting sites shown in our study may act as stopovers, or be used seasonally to reduce commuting to feeding sites.

Adults of both sexes travelled longer distances in winter than in summer: this could be the consequence of reproductive demands on females in summer (although no females were obviously pregnant or had dependant young at the time of capture, some may have given birth later). Between October and February, while females care for their pups, males may form ‘bachelor’ colonies (K. Ruhomaun, pers. com.). That would suggest that females form maternity colonies as for example in *P. vampyrus natunae* in Sarawak [55]. However, long-term monitoring of major roosting areas and a detailed analysis of roost switching behaviour are necessary to confirm such a pattern. Alternatively resources could be scarcer in winter, and bats may need to travel further to find them. We found no overall difference in the nightly distances covered by males and females. In Madagascar *P. rufus* females travelled significantly longer distances than males (average 28.1 km and 15.4 km respectively) [13]. Banack and Grant [55] found that juvenile male *P. tonganus* travel on long, so called ‘exploratory flights’ of up to 46.7 km. We found that immature male *P. niger* travelled further per night in summer than in winter. This could be associated with dispersal (perhaps involving ‘exploratory flights’, as males are the dispersing sex in a wide range of mammal species) [56].

Other *Pteropus* species also show seasonal movement patterns. For example, Australian *P. scapulatus* makes seasonal movements in response to flowering and fruiting of their food plants [57]. *Pteropus tonganus* changes roosting sites in response to food availability [58]. In contrast, Bonin flying foxes *P. pselaphon*, exhibit seasonal changes in roosting patterns associated with the breeding cycle. They form colonial roosts in winter and dispersed roosts (more roosting sites with less individuals roosting together) in summer. In this case, roosting facilitates social interactions for mating and potentially also information exchange [48]. Our roosting observations suggest that Mauritius fruit bats form colonial roosts during the winter in the southern part of the island and roost more in the northern part during the summer. This pattern could be associated with the remaining forest cover. The Black River Gorges National Park in the south-west offers some of the last remaining primary forest fragments which may provide substantial quantities of food for the bats during winter. On the other hand, during the summer, the majority of both native and introduced plant species are fruiting across the island and the north of Mauritius is usually the first to bear native as well as commercial fruits such as mango or lychee due to its warmer climate [59]. That may explain the shift in distribution patterns.

Our results provide new information that has relevance for the conservation and management of *P. niger*. The recent culls have had a major impact on *P. niger* populations on Mauritius, and have been highly controversial [60]. None of the tagged bats disappeared during the 2015 organised cull, suggesting that the eight bats from which we lost signal were poached by local people. Hence the effects of local poaching may be substantial. For effective conservation of *P. niger*, broader conservation initiatives involving a number of stakeholders are needed [61, 62]. Increased use of netting to protect fruit will minimise conflict between fruit growers and bat conservation [28]. Culling may force bats into new areas where they were previously not eating commercial fruit [63]. Indeed, the recent culls have failed to increase commercial profits at fruit farms [64]. Our data provide context by showing that the bats cover very large distances and any effects of culling will not be localised and may have little impact over broad spatial scales.

## Conclusion

*Pteropus niger* is similar to other *Pteropus* species and can travel long distances during the night in search of food and for social and mating interactions. The home range of the species is the whole island of Mauritius and bats frequently move between the roosts and remnant forest fragments that are important for bat conservation. The vagility of the species explains its panmictic genetic structure. At present the extent of the cull is of major concern given that over 95% of native forest has been lost since 1638 [65, 66] and remaining native forest patches are being degraded by invasive plant species that may affect the availability of native fruit species [65]: the Mauritian flying fox plays a vital role in the dispersal of native and endemic plant species [20]], and probably in enhancing seed germination and potentially forest regeneration over large spatial scales as occurs with another endemic flying fox species (*Pteropus rufus*) in nearby Madagascar [14]. The role of *P. niger* as a seed disperser is especially important at present, as it is the largest extant seed disperser on Mauritius following the extirpation of megafauna such as giant tortoises [67]. The cull is currently based on a lack of scientific evidence [68]. Although *P. niger* can move large distances in short time periods and can therefore respond rapidly to threats and environmental change over small spatial scales, it is effectively ‘trapped’ on a small island where it is difficult to escape the effects of mass culling. Culling is unlikely to have localised benefits for reducing damage at orchards given the extensive movements of the bats.

## Additional file

Additional file 1: **Table S1.** Description of the top five most parsimonious GLMMs built to test whether nightly movement of *Pteropus niger* differs between sex (male vs. female), age (sexually mature vs. immature), and season (summer vs. winter). Models are ranked in ascending order of *AICc*. The number of parameters (*K*), *AICc* weight (*Wt*), and cumulative weight (Cum*. Wt*) are given for each model. The model that includes the three-way interactions was considered as the best one. **Table S2.** Results of the post hoc contrast tests applied to the most parsimonious GLMM that included the three-way interactions between sex (male vs. female), age (sexually mature vs. immature), and season (summer vs. winter) for explaining nightly movement of *Pteropus niger*. Estimate with associated standard error (SE) as well as *Z* ratio and adjusted *P*-value are given for each comparison. (DOCX 18 kb)

## Abbreviations

F: Female
GPS: Global Positioning System
GSM: Global System for Mobile Communications
IUCN: International Union for the Conservation of Nature
M: Male
NPCS: National Parks and Conservation Services
SI: Sexually immature
SM: Sexually mature
