# Supplementary material for: The movement ecology of the Mauritian flying fox (Pteropus niger): a long-term study using solar-powered GSM/GPS tags
Source: Mov Ecol. 2019 Apr 15;7:12. doi: 10.1186/s40462-019-0156-6 (PMC6463659; doi:10.1186/s40462-019-0156-6)
Supplement: Supplementary file 1 — Table S1. Description of the top five most parsimonious GLMMs built to test whether nightly movement of Pteropus niger differs between sex (male vs. female), age (sexually mature vs. immature), and season (summer vs. winter). Models are ranked in ascending order of AICc. The number of parameters (K), AICc weight (Wt), and cumulative weight (Cum. Wt) are given for each model. The model that includes the three-way interactions was considered as the best one. Table S2. Results of the post hoc contrast tests applied to the most parsimonious GLMM that included the three-way interactions between sex (male vs. female), age (sexually mature vs. immature), and season (summer vs. winter) for explaining nightly movement of Pteropus niger. Estimate with associated standard error (SE) as well as Z ratio and adjusted P-value are given for each comparison. (DOCX 18 kb) [file 40462_2019_156_MOESM1_ESM.docx]

**Table S1.** Description of the top five most parsimonious GLMMs built to test whether nightly movement of *Pteropus niger* differs between sex (male vs. female), age (sexually mature vs. immature), and season (summer vs. winter). Models are ranked in ascending order of *AICc*. The number of parameters (*K*), *AICc* weight (*Wt*), and cumulative weight (*Cum. Wt*) are given for each model. The model that includes the three-way interactions was considered as the best one.

| **Model** | ***K*** | ***AICc*** | ***ΔAICc*** | ***AICc Wt*** | ***Cum. Wt*** |
| --- | --- | --- | --- | --- | --- |
| age*season*sex | 10 | 10028.30 | 0.00 | 0.78 | 0.78 |
| age*season + sex | 7 | 10032.40 | 4.12 | 0.10 | 0.88 |
| age*season + season*sex | 8 | 10033.90 | 5.60 | 0.05 | 0.92 |
| age*season + age*sex | 8 | 10034.00 | 5.74 | 0.04 | 0.97 |
| age*season | 6 | 10034.60 | 6.33 | 0.03 | 1.00 |

**Table S2.** Results of the post hoc contrast tests applied to the most parsimonious GLMM that included the three-way interactions between sex (male vs. female), age (sexually mature vs. immature), and season (summer vs. winter) for explaining nightly movement of *Pteropus niger*. Estimate with associated standard error (SE) as well as *Z* ratio and adjusted *P*-value are given for each comparison.

| **Contrast** | | **Estimate**  **(± SE)** | ***Z* ratio** | ***P*_adj_** |
| --- | --- | --- | --- | --- |
| ♀ mature summer vs. | ♀ mature winter | -0.69 (± 0.19) | -3.74 | ** |
|  | ♂ mature summer | -0.57 (± 0.68) | -0.84 | NS |
|  | ♂ mature winter | -1.58 (± 0.71) | -2.22 | NS |
|  | ♀ immature summer | 1.16 (± 0.71) | 1.63 | NS |
|  | ♀ immature winter | 0.14 (± 0.72) | 0.20 | NS |
|  | ♂ immature summer | -0.64 (± 0.55) | -1.15 | NS |
|  | ♂ immature winter | -0.30 (± 0.56) | -0.54 | NS |
| ♀ mature winter vs. | ♂ mature summer | 0.13 (± 0.68) | 0.19 | NS |
|  | ♂ mature winter | -0.89 (± 0.71) | -1.25 | NS |
|  | ♀ immature summer | 1.85 (± 0.71) | 2.60 | NS |
|  | ♀ immature winter | 0.84 (± 0.71) | 1.17 | NS |
|  | ♂ immature summer | 0.06 (± 0.55) | 0.10 | NS |
|  | ♂ immature winter | 0.40 (± 0.56) | 0.71 | NS |
| ♂ mature summer vs. | ♂ mature winter | -1.02 (± 0.25) | -4.05 | ** |
|  | ♀ immature summer | 1.72 (± 0.81) | 2.13 | NS |
|  | ♀ immature winter | 0.71 (± 0.81) | 0.87 | NS |
|  | ♂ immature summer | -0.07 (± 0.67) | -0.11 | NS |
|  | ♂ immature winter | 0.27 (± 0.68) | 0.40 | NS |
| ♂ mature winter vs. | ♀ immature summer | 2.74 (± 0.84) | 3.27 | * |
|  | ♀ immature winter | 1.73 (± 0.85) | 2.04 | NS |
|  | ♂ immature summer | 0.94 (± 0.71) | 1.33 | NS |
|  | ♂ immature winter | 1.28 (± 0.71) | 1.80 | NS |
| ♀ immature summer vs. | ♀ immature winter | -1.01 (± 0.50) | -2.05 | NS |
|  | ♂ immature summer | -1.80 (± 0.71) | -2.55 | NS |
|  | ♂ immature winter | -1.45 (± 0.71) | -2.05 | NS |
| ♀ immature winter vs. | ♂ immature summer | -0.78 (± 0.71) | -1.10 | NS |
|  | ♂ immature winter | -0.44 (± 0.72) | -0.62 | NS |
| ♂ immature summer vs. | ♂ immature winter | 0.34 (± 0.10) | 3.39 | * |

NS: *P*_adj_ ≥ 0.05; * *P*_adj_ < 0.05; ** *P*_adj_ < 0.01
